# Zebrafish Xenograft: An Evolutionary Experiment in Tumour Biology

**DOI:** 10.3390/genes8090220

**Published:** 2017-09-05

**Authors:** Rachael A. Wyatt, Nhu P. V. Trieu, Bryan D. Crawford

**Affiliations:** Department of Biology, University of New Brunswick, Fredericton, NB E3B 5A3, Canada; r.a.wyatt@unb.ca (R.A.W.); ntrieu@unb.ca (N.P.V.T.)

**Keywords:** xenograft, zebrafish, extracellular matrix, matrix metalloproteinases, MMPs

## Abstract

Though the cancer research community has used mouse xenografts for decades more than zebrafish xenografts, zebrafish have much to offer: they are cheap, easy to work with, and the embryonic model is relatively easy to use in high-throughput assays. Zebrafish can be imaged live, allowing us to observe cellular and molecular processes in vivo in real time. Opponents dismiss the zebrafish model due to the evolutionary distance between zebrafish and humans, as compared to mice, but proponents argue for the zebrafish xenograft’s superiority to cell culture systems and its advantages in imaging. This review places the zebrafish xenograft in the context of current views on cancer and gives an overview of how several aspects of this evolutionary disease can be addressed in the zebrafish model. Zebrafish are missing homologs of some human proteins and (of particular interest) several members of the matrix metalloproteinase (MMP) family of proteases, which are known for their importance in tumour biology. This review draws attention to the implicit evolutionary experiment taking place when the molecular ecology of the xenograft host is significantly different than that of the donor.

## 1. Introduction

The zebrafish xenograft is a model for tumour biology that has grown in popularity in the last decade, most often used to test drugs for their cytotoxic, anti-metastatic, or anti-angiogenic properties, but also as a more sophisticated alternative to 2D culturing assays that use artificial matrix or matrix extract to investigate cellular invasiveness. Zebrafish embryos are much easier to image than mice, the most prominent xenograft model for studying cancer, and they allow for better analysis of the molecular components required for invasion [1,2,3]. Zebrafish are better suited to high throughput approaches than mice while still having the advantage of an in vivo extracellular matrix (ECM) that provides epitopes and intramolecular forces that mimic more closely the variation of tissues in humans. Though 2D/3D culturing and other in vitro assays are an important step in asking questions about the effect of ECM molecules and forces on cancer cells, the zebrafish xenograft provides a good compromise: the evolutionary distance between zebrafish and humans is larger than that between mice and humans, but the zebrafish offers a versatile model for in vivo ECM that has clear advantages over matrix extracts like matrigel or artificial hydrogel matrices. Other perspectives on the advantages of the zebrafish xenograft model are reviewed in [4,5,6,7].

Cancer is a complex evolutionary and ecological disease [8,9,10,11], in that tumour cells are genetically variable, and their interactions with each other and their tissue microenvironment (TME) provides a complex selection landscape. This disease must therefore be treated differently from many other pathologies because of the variation in driver mutations [12] and heterogeneity of mutations and cell phenotypes. Much cancer research is focused on the urgent need for drugs to combat advanced forms of the disease, and different researchers take different angles when looking for drug targets [13]. Drug discovery research falls roughly into four camps that each employ a specific strategy toward combating a few of the major hallmarks of cancer. One strategy is the development of cytotoxic drugs that aim to target quickly dividing cell populations disproportionately [14]. Alternatively, anti-angiogenic drugs cut off resources in order to starve the tumour [15], while anti-metastatic drugs limit the ability of the cancer to spread [16]. Researchers also work to find ways to sensitize the immune system to the quickly evolving tumour cells, taking advantage of the body’s natural ability to fight infection [17]. Drug treatment strategies are often applied in the clinic as cocktails to lower the chance of cancer resistance and escape. Cancer cells and their microenvironment can be viewed through an ecological, as well as evolutionary, lens. While there are markers and characteristics common to many subtypes of cancers, each cancer is a unique ecosystem of interacting parts. Tumours evade immune monitoring, natural control over growth, and tissue boundaries through many molecular pathways, and there are tradeoffs and compensatory mechanisms that allow cancer cells to escape death in many cases. In the same way that an ecosystem buffers and adapts to change to varying extents, so too do developing cancers. 

This review will give an overview of cancer and the cancer microenvironment as an ecological and evolutionary disease in order to highlight some advantages and disadvantages of using zebrafish xenograft models. Xenografts may help to ask questions about the principles of the ecology of cancer in the same way that invasive species in a new environment can provide insight into ecological mechanisms that are important in its control [18]. The zebrafish xenograft, both despite and because of its evolutionary distance from mammals, can offer insights into the mechanisms associated with cancer progression. We draw explicit attention to the experiment performed when molecular components involved in cancer progression are absent in the host. Gaining a better understanding of how mechanisms are conserved from species to species will also lead to a better frame of reference for how they are conserved from cancer to cancer. 

## 2. Cancer Context

The ECM has a critical role to play in many of the processes of cancer as the substrate to which cells attach and respond. Metastasis is the single greatest cause of cancer deaths [19] and as a result is a strong candidate for clinical intervention. Mechanisms of metastasis are more complex than the simplistic picture of a cell migrating through matrix to a new location. Traditionally, metastasis is characterized as a cell’s migration through the basement membrane, intravasation, circulation, immune evasion, extravasation, and then colonization. This stepwise progression turns out to be an oversimplification, as many steps of metastasis may be happening at the same time [19,20]. Migrating cells must move through the ECM substrate during cell migration and invasion [21]. The ECM may sequester growth factors (such as fibroblast growth factor-2, which bind heparan sulfate proteoglycans in the matrix), releasing them as degradation products, and can itself provide signals in the form of some of its components: laminin and tenascin-C bind epidermal growth factor receptors to stimulate growth [22]. Signalling sites can be exposed during degradation, and the breakdown products of most, if not all ECM components can also be signalling factors (reviewed in [23]). Cells are sensitive to physical force, and mechanical load on the ECM can expose cryptic ligands [24]. A stiffer matrix can in and of itself stimulate a cell to undergo an epithelial to mesenchymal transition and begin to migrate [25,26], and there is evidence that the correlation between stiffness and tumour progression also applies in the natural tumour microenvironment [27,28]. Traditional in vitro assays for migration that use a matrix extract or artificial matrix is as a substrate through which cells may or may not migrate may under- or overestimate the invasive potential of some cells that would invade under conditions that might be found in the tumour itself. The xenograft has the advantage of a native, functional ECM, which is a step closer to emulating the components and stiffness in the normal TME, though the location of the xenograft will not have any of the cooperative conditioning that the native environment of the tumour would have.

### 2.1. Structure of the Tumour Microenvironment 

The microenvironment surrounding cancer cells is modified by the developing tumour to enhance the survival of the tumour cells [29,30] by, for example, the induction of chronic inflammation through signals such as transforming growth factor β (TGF β), tumour necrosis factor (TNF) or one of many interleukins [31] and angiogenesis by vascular endothelial growth factors (VEGFs), hypoxia inducible factor-1 (HIF-1), and notch signalling [32]. Though historically cancer was viewed as a tissue-based maladaptive response compounded by inflammation [33], the majority of recent literature has been focused on key molecular players such as TGFβ [34], VEGF [35], and matrix metalloproteinases (MMPs) [36]. This paradigm views cancer as a cell-mediated disease, but we are now starting to return to a more integrated model of cancer as a whole tissue, rather than just focusing on its individual components.

The TME is more heterogeneous than was once thought. Phenotypic and functional heterogeneity of the cells participating in the cancer microenvironment contribute to the complexity of the TME [37,38], and the TME varies substantially from the core of a tumour to its periphery, with different densities of stromal cells, lymphocytes and variously sized vessels, and different phenotypic populations of the cancer cells themselves [11,39]. The sub-clonal heterogeneity of the tumour has a major effect on the phenotype, where minor subpopulations can be major drivers of tumour growth [40]. Presumably, the same principle can drive the structure and function of the TME, but little is known about how sub-clonal cell populations contribute to the basic structure of the ECM. While we have begun to map spatial heterogeneity to increase our understanding of how these cells interact with each other and their environment, this has largely been focused on the organization of non-tumour cells [39] or subpopulations of tumour cells [11]. Currently, we study the TME largely through the lens of angiogenesis, immune modulation or cell morphology, with the latter having not been revisited since the end of the twentieth century. Angiogenesis in the TME results in vessels that lack multiple basement membrane proteins, have regions of thin endothelium and small gaps between endothelial cells [41], and endothelial-like cells that can form structures similar to small capillaries in vasculogenic mimicry [42]. Extravasation and metastasis is facilitated by breaches in endothelial barriers induced by VEGF [43]. While some studies do correlative work to look at key molecular players in the context of ultrastructure [41], the majority of them do not. None look at the distribution of key molecular players in the context of ultrastructure, which could help explain spatial heterogeneity in ways that other structures such as blood vessels and cells have not.

Ultrastructural examinations, which would aide in mapping the components of the ECM, have not been updated recently. The bulk of broad view surveys were done during the late twentieth century using electron microscopy (EM), and even then, only a few were done on xenografts. Serial xenografts maintain their mitotic activity and retain characteristics of the tumours of origin, but they exhibit different levels of necrosis and a reduction in stromal tissue [44,45,46]. Major structural differences, such as a lack of desmosomes or other cell junctions and large extracellular deposits of electron dense material, can also be present depending on the level of differentiation [45]. Even within one histopathological class of germ cell tumour, there is marked ultrastructural heterogeneity between tumours [45]. For some tumours, even when there is a relatively uniform histological appearance, other biological markers reveal that the tumours are very clearly heterogenous. Tumours have highly variable vascular distribution and density, with chaotic arrangements that are at times leaky and incomplete, and often have missing basement membranes and necrosis independent of spatial organization [47]. Revisiting the ultrastructure of the TME using modern tools, especially in the context of xenografts in which specific components can be up- or down-regulated and/or epitope tagged, will likely yield important insights into the mechanisms at play in the host/tumour interface and how these may be employed to clinical advantage. 

Techniques such as immunogold, correlative EM, 3D reconstruction with serial electron microscopy, and focused ion beam scanning electron microscopy can allow us to make spatial connections between molecular players in the TME and the ultrastructure. The zebrafish xenograft model, which allows for easy tracking of tumour cells [6], will enable more precise and higher throughput examinations of the TME and can be used to compare ultrastructure and heterogeneity of molecular components in both primary and secondary locations. Much as the chick-quail and other chimeric embryos employed by classical developmental biologists were so effective in the analysis of gastrulation, organogenesis, and the invasive behaviours of neural crest cells [48], using species-specific antibodies, zebrafish xenografts will allow us to unequivocally determine which cells are contributing which molecules to the TME, when and where they are deposited, and potentially how these molecules are being modified by the activities of various cell types. We have the technology to ask how far signaling molecules and other extracellular effectors such as proteases diffuse, how expression is initiated and spread from the initial immediate microenvironment, and how these processes and components are integrated in three-dimensional space in vivo. However, to our knowledge, these questions are not being addressed using the xenografting approach.

### 2.2. Migration and Invasion

The most basic requirement of metastasis is migration and invasion of cancer cells. These cells co-opt mechanisms of migration and invasion from normal developmental and homeostatic processes. For example, cancer development often involves genes controlled by STAT3 signalling, known for its role in wound healing [49], while the epithelial to mesenchymal transition of migratory neural crest cells has similar expression patterns to malignant cancer cell populations [50]. The extracellular matrix components, integrins, and MMPs involved in cancer show patterns similar to those required for implantation [51]. Xenografting provides an ideal system in which to investigate the extent to which these changes are occurring within “normal” tissues surrounding a tumour and how the tumour induces these changes. 

Treating a tumour with drugs that are either cytotoxic or sensitize the cells to their toxic local environment puts a stronger selective pressure on them to evade death. Inhibiting some aspects of tumour progression may in fact increase the rate of metastasis. In the ideal case of completely blocked vessel formation into a tumour with a physical barrier, cancer cells still migrate and form distant metastases and in highly metastatic tumours may metastasize more than tumours connected to the host vasculature [52]. Development of preventative treatments that are non-lethal to the cancer cells themselves may be beneficial [9,53] by encouraging slow life histories as opposed to fast life histories [54]. Having a stronger grasp of the ecological and evolutionary forces in a tumour will allow us to rationally design treatments that take advantage of trade-offs associated with the adaptations made by tumour cells.

Models of cell migration during the development of a malignant tumour need to incorporate the varied modes of invasion and migration while taking into account the local environment. Cells may individually secrete the necessary components and signals in their local environment through specialized structures such as invadopodia [55,56,57], then pull themselves along through degraded matrix and cell debris to move through epithelia and basement membranes, or cells may migrate cooperatively as a unit through barriers [58,59,60]. Once neovascularization has started, however, the layers of normal tissue are perturbed. Cells have access to more resources through the nascent vascular system, but the epithelia of these vessels are usually highly disorganized and leaky [20]. In the context of a native tumour cell population, cells may escape into the vascular system without the need for breaching the basement membrane and epithelia of the vasculature. In fact, circulating tumour cells are consistently found in pre-metastatic patients and may in some instances prove a useful diagnostic feature of the disease [61,62]. Regardless, a stepwise view of metastasis is a problematic oversimplification, and metastasis may be better addressed by in vivo investigations.

Cancer evolves in the context of communication networks present in its originating tissue. The specific cell-cell communication between tumour cells and their environment is an important aspect of this disease. Surrounding stromal cells are a source of chemokines that enhance proliferation and migration [63] and can secrete many factors that modulate the immune response [64]. Tumour-associated fibroblasts may also contribute the proteases and signals required for migration or even lead collective migration [65]. Any model that only makes use of the cancer cells themselves will miss important factors in disease development and possible opportunities for drug development. 

A tumour generates complex interactions with its local environment and is itself comprised of a non-homogeneous set of wildly varying cell types. In experiments with a mix of two cell types, one that is invasive alone and one that is not, both cell types ended up migrating when grafted in combination. In co-culture grafts, cells from the migratory cell line were generally found at the tip of invading groups of cells comprised of a mix of cells from the two lines [59] in a similar fashion to how tumour associated fibroblasts can lead collective cell migration in some cases [65]. Grafts of the migratory line alone were insensitive to protease inhibitors, but in co-culture grafts, the migration of both cell lines could be affected by protease inhibitors [59]. The interactions between different tumour cell subpopulations cannot be overlooked.

### 2.3. Metastasis: Beyond Migration and Invasion

In order for a cancer to metastasize, it needs to disperse its cells by migration and invasion into new tissues and it also needs to colonize a new location. Cancer is usually thought to invade new tissues via the circulatory system, but may also disseminate via the lymphatic system or through solid tissues. An increasing number of cancer researchers are interested in what makes cells capable of creating a de novo tumour at a new location. A tumour is a varied population of cells that consists of many evolutionary dead-ends. Transplantation studies show that not all tumour cells are capable of initiating a new tumour [66]. Circulating tumour cells can be detected in the blood in many forms of the disease and may be useful in predicting malignancy [67], but not many of these cells can initiate tumours. Cells that have the ability to initiate a tumour are termed cancer stem cells (CSCs) or tumour initiating cells (TICs) and are characterized by their phenotypic plasticity (from growth capabilities to nutritional paradigms, from epithelial to mesenchymal characteristics) and ability to grow a new tumour [68]. The locations of metastases are not random: certain cancer types are known to establish metastases in characteristic locations (e.g., breast cancer to lung, bone, and liver). These preferential “niches” may provide important clues about the mechanisms of cancer metastasis, and the conditioning of locations of downstream metastases may be an important step in development of the disease. Some have suggested that metastatic locations are pre-conditioned by soluble factors and/or circulating tumour cells that are either incapable of colonization or fail at colonization. Along with passive processes like filtration through capillaries, these niches may direct characteristic patterns of metastasis [69].

The xenograft may serve well as a model for investigating CSC competency but is not as well equipped to answer questions about how metastatic locations may undergo pre-conditioning by secreted proteins or precursor cells before colonization by metastatic cells. The zebrafish xenograft may provide insight into evolutionarily conserved aspects of niche preference, as there is evidence that different cancer cells target different areas for their secondary metastases in the zebrafish [70]. Conserved molecular features, or those which are more evolutionarily derived, may provide clues to this targeting process. The xenograft is, however, poorly prepared to ask questions about cancer’s development with its microenvironment and stroma, as it generally combines cells from a developed cancer with a naïve ECM. Instead, the model may be better prepared to ask how cancer cells might behave at a metastatic location.

## 3. Cancer out of Context

### 3.1. Simulating the Microenvironment

To avoid the downsides of studying individual cancer cells on 2D surfaces, several strategies are employed. First, simply using more cell lines will avoid overinterpretation of some of the altered behaviour of individual cell lines [71]. Cells grown under traditional media with or without serum may not maintain the characteristics of their original tumours, but may be affected by very small changes in pH or growth density, and so need to be carefully controlled [72]. Some cells grow and behave differently in 2D culture: endometrial cancer cells change growth patterns, secrete different soluble signals and exhibit altered metabolism when compared to 3D cultured cells [73], and leukemia cells are differentially sensitive to chemotherapy when cultured in a 3D [74]. This is not an isolated phenomenon—for other examples, see [75,76,77,78]. The matrix in 3D cultures is ideal for simplifying the ECM in order to ask questions like what effect the stiffness or the specific molecular composition of the matrix has on migration, but it is not a replacement for in vivo studies. 

The varied ECM of real tissues are much more difficult to model, which is another advantage of xenografting. An in vivo ECM allows cancer cells to communicate with the microenvironment and have access to many different types of matrix. Commonly used xenograft models transplant human cancer cells into immunocompromised mice, the chorioallantoic membrane (CAM) of developing chicks, or into various tissue contexts within zebrafish embryos. Primary small-cell lung carcinoma cells grown in a serial xenograft express different genes than a parallel set of cells cultured traditionally in dishes and reintroduced into a xenograft, and authors suggest that this could be occurring with many cell lines [79]. Given that the cancer microenvironment is a significant part of the pathology, it is important to think about the relevance of grafting and transplant studies that necessitate a surrogate stroma that will be different from the native context of the cancer. There are many strategies used to minimize the effect of these approximations. The first is to use mice: searching an abstracting database for mouse xenografts will yield orders of magnitude more publications per year than zebrafish or CAM xenografts. Because mice are more closely related to humans, it is reasonable to suppose that their tissues are more similar at the molecular level, and that the behaviour of xenografted human tumour cells will therefore be more representative of their behaviour in a human patient. A second strategy is to transplant the cancer cells into the tissue most closely related to their origin tissue. Transplantation studies in which human mammary cells are transplanted into mouse, however, show that there are some important differences in the microenvironment [80]. To get around this, some labs are working to “humanize” the transplant host using approaches ranging from expressing human cell markers in a given tissue to transplanting normal human tissue culture cells in the host along with the cancer cells [80,81]. We need to ask how much of their behaviour is conserved when cells are put into an evolutionary divergent context, such as the zebrafish xenograft.

Beside the problems already associated with working with cell cultures [82,83], drug development using cancer cell lines frequently yields compounds that fail phase III clinical trials [84,85]. We have known for a long time that cell lines behave differently once they have been cultured, as exemplified by this study from the 1980s comparing suite of melanoma cell lines [86]. Cancer cell lines may no longer maintain the characteristics of the original tumours and they lack critical elements from the microenvironment. Wilding and Bodmer articulate the current opinion in the field that cell lines are a big part of the disconnect between translational research and clinical research and suggest ways that cell culture and xenograft models can be improved [85]. Even between different species of grafting hosts, however, there are examples of discrepancies in drug responses, possibly as a result of the bioavailability of tested drugs [87]. This discrepancy highlights the need to use many different models and primary cells whenever possible to get the best representation of the disease. One very promising use of the zebrafish xenograft, reviewed elsewhere [4,88], is as a clinical tool to test the efficacy of drug cocktails on patient samples to avoid treatment with ineffective chemotherapeutics that might end up making the disease worse. This model needs to be carefully interpreted to best translate drug dosage into clinical research.

### 3.2. The Immune Problem

The immune system has various effects on the growth and progression of a tumour. The chronic inflammation often associated with tumours recruits immune cells that can stimulate angiogenesis [33]. Anti-inflammatories may be an important addition to existing anti-angiogenic strategies, as a way of circumventing immune-mediated angiogenesis. The adaptive immune system, however, can monitor cells for aberrant antigens, making immune evasion an important precondition to a developing tumour’s success [89,90]. Immune sensitization is one technique used in coordination with other drugs and treatments during chemotherapy [17,91,92,93]. Tumours have a dynamic relationship with tumour-infiltrating lymphocytes (TILs) throughout their development. TILs are markers for good prognosis in many cancer types, and it has been suggested that the presence of lymphocytes may represent a defense against cancer progression. Immune sensitization strategies have been renewed in earnest because of the strong association between TILs and disease outcome [94]. 

One of the real negatives to using any xenograft is the role that the immune system has in cancer development. Even in a very similar microenvironment (such as that within humanized mice), the immune system of the mouse must be impaired in order for the xenograft to take hold, grow, and proliferate. Likewise, with CAM and zebrafish xenografts, generally grafts are done at stages where the immune system has not yet fully developed. Allografts, especially syngeneic allografts, provide some alternatives that allow mature tumours to be grafted into organisms with fully competent immune systems [95]. Immune rejection is less likely for genetically identical individuals. Cancer cells may still end up diverging enough to provoke an immune response once they are taken out of their native context. The communication between cancer cells and the host of origin’s immune system builds over the lifespan of the tumour. A novel alternative being developed in zebrafish is to pre-seed the host with irradiated cancer cells in order to promote immune tolerance early in development, then doing grafts later in the host’s life, where the cancer cells will not immediately raise a humoral immune response [96]. Zebrafish are an ideal model for this work, as they can easily be injected during embryonic stages before the humoral immune system has full developed, and pigment-free strains allow easy imaging [97,98].

## 4. Zebrafish Xenograft: An Evolutionary Experiment

Transplanting cells from one organism to another is an evolutionary experiment: for example, the factors required for survival and disease progression, or the elimination of tumour cells, may be absent or unrecognizable in the new microenvironment. It is important to remember that injection of human cancer cells into any host (including mouse) will impair the ability of the cancer cells to communicate with the stromal cells in their new environment. An interesting consequence of doing trans-species grafting is that the molecular components from the host (representing the tumour microenvironment) and the cancer cells can be differentiated by species-specific antibodies. Interpretation is non-trivial for host species that display different complements of proteins as a consequence of, for example, gene loss or duplication events. Zebrafish are part of the teleost radiation that followed a whole genome duplication in the common ancestor to the teleosts [99]. Many gene clusters in zebrafish are duplicated when compared to their mammalian counterparts, while others have been lost. Similar patterns exist for other model fish whose genomes have been sequenced [100,101,102]. These missing and/or duplicated genes may have interesting effects on the behaviour of grafted cells and need to be taken into account during interpretation. 

### 4.1. Case Study: Matrix Metalloproteinases

**Figure 1:**
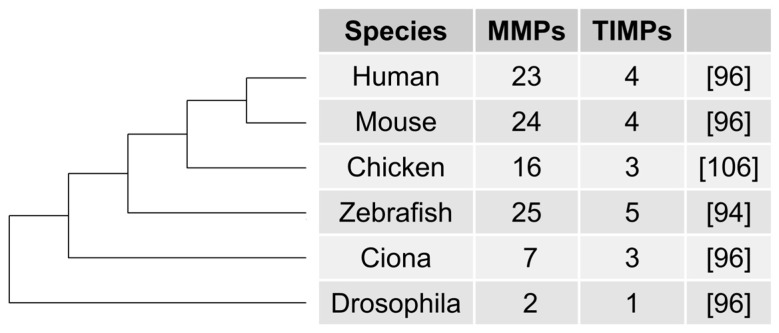
Matrix metalloproteinase (MMP) and tissue inhibitor of matrix metalloproteinase (TIMP) representation across common model species from the literature.

The evolutionary history of the ECM and its modulators is tightly tied to the origins of multicellularity, as is cancer. Its components form the structure and support for cells that make up tissues and organs, and in its original conception was considered a static structure that holds tissues together. Decades of work have shown that it is more than simply a static structural component. The ECM is dynamic and has both mechanical and signalling roles [103,104]. The MMPs are a family of proteins that were named for their ability to degrade the otherwise proteolytically resistant ECM components. The family has continued to grow in size, with upwards of two dozen described members currently [105]. The complexity of the metzincin family of proteases (of which MMPs are a part) seems to increase with organism complexity: *Drosophila melanogaster* and *Ciona intestinalis* have less than 10, while vertebrates maintain closer to two dozen (Figure 1). This numerical trend is consistent with their endogenous inhibitors, the tissue inhibitors of matrix metalloproteinases (TIMPs). MMPs were initially studied in the hopes of developing inhibitors that could block metastasis. It turns out that broad-spectrum MMP inhibitors are not an effective treatment option, though many MMPs have been implicated in poor prognosis and cancer progression [106,107,108,109,110,111,112,113]. More focused targets are required in order to make use of their unique roles in cancer development [114,115]. 

The MMPs have been pursued as possible targets for chemotherapy because of their roles in degrading ECM barriers between tissues, stimulating angiogenesis, and releasing sequestered growth factors. Unfortunately, clinical studies using general MMP inhibitors have been ineffective. Because there are so many members in this family of proteins, it is hard to know which may be most relevant to cancer progression. They are secreted as inactive zymogens and so are difficult to study because analysis of expression does not necessarily correlate with activity in time or space. Furthermore, they may only become activated under specific conditions within ECM, underscoring the importance of in vivo studies. Various authors have suggested that the most relevant targets are the activator proteins, and the focus here has been largely on activators like plasmin [116,117,118] or MT1-MMP (also known as MMP14). Root activators are difficult to trace in cases of proteolytic activation cascades, and so the question of what activates the effectors may be a less effective strategy for designing drug targets than simply targeting the downstream effectors. 

**Figure 2:**
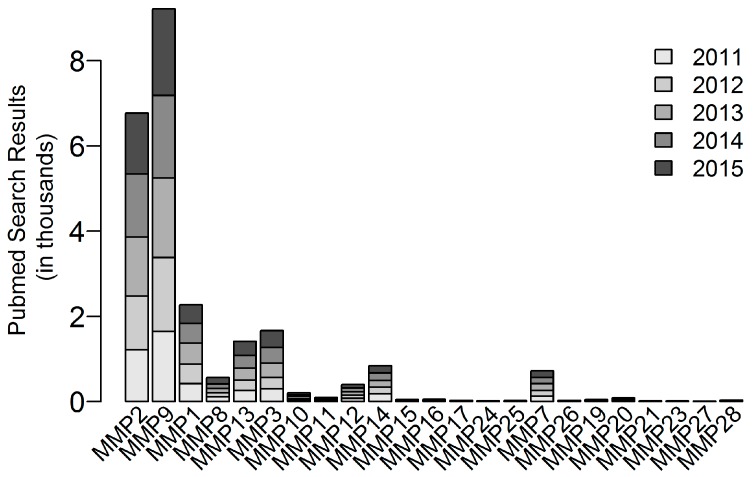
Literature distribution by keyword searches for the several most common names of the family of MMPs. Scale represents 0–10,000 hits in PubMed.

Most screens will choose a small subset of MMPs to assay, and as a result, most mechanistic studies are focused on several highly studied MMPs. The most studied MMPs are MMP2 and MMP9: they were among the earliest discovered, and their activity can be assayed relatively easily by gelatin zymography. Each of MMP2 and MMP9 are mentioned almost more individually in the literature than the sum of all the rest of the MMPs (Figure 2). Similarly, MMP14 (MT1-MMP) is the focus of most studies on membrane type MMPs. Other MT-MMPs share homology and could have similar roles to MT1-MMP as activators, but are much less likely to be screened for, let alone studied mechanistically. Various lesser studied MT-MMPs are involved in migration mechanisms during development. For example, Mmp17b (MT4-MMP) is required for proper neural crest migration [119], and Mmp25 (leukolysin, MT6-MMP) is involved in axon pathfinding during the development of the zebrafish nervous system [120]. Cancer cell invasion often makes use of mechanisms found in normal development and tissue homeostasis [49,50,51], but these MMPs are not commonly screened for in cancer research. 

Another advantage of the zebrafish xenograft model is its amenability to in vivo analysis of MMP activity [121]. The changes in MMP activation and activity associated with xenografted cells could be characterized directly in the context of the zebrafish embryo, although to our knowledge, this has not been done. Narrowing down which MMPs may be involved most heavily in migration and invasion is a challenge that needs to be overcome before drug targeting efforts will result in successful treatment options.

### 4.2. Xenografting as an Evolutionary Experiment 

The zebrafish xenograft is a more complex experiment than a mouse xenograft because of the increased genetic difference between host and graft. For example, zebrafish are missing many of the MMPs commonly associated with cancer (see Table 1), several of which have value as poor prognosis factors. Several of the missing members of the MMP family (MMP1, 3, 7, 10, and 12) are also expressed in the reproductive system [122,123], and may have mammalian-specific roles in implantation, gestation and endometrial function. While it is important to remember that there are significant differences between the zebrafish and mammalian systems (differences that exist to a smaller extent between mice and humans) that may cause cells to behave differently in a mouse xenograft from a zebrafish xenograft, these same differences may allow us to ask questions about the contributions of the microenvironment to the behaviour of cancer cells. Xenografting is commonly used as a way to quickly test manipulations of cells in an in vivo context. For example, phosphatase and tensin homologue (PTEN) deletion can increase the invasive behaviour of MCF-7 breast cancer cells [124], MMP9 expression is correlated with invasiveness [70,125], and blocking invasion using several drugs downregulates MMP expression [70,126] in the zebrafish xenograft model. Induced models of cancer exhibit a correlation between increased migration and the level of MMP expression [125]. 

Single manipulations may provide some leads to what players and pathways are the most important, but much more powerfully: because zebrafish are missing many members of various gene families, the zebrafish xenograft can be viewed as an evolutionary experiment that will enable us to answer questions about conserved mechanisms between vertebrates, and in particular, which molecules encoded by the genome are most important in cancer progression. Few comparisons exist comparing zebrafish to mouse xenografts except where they are used to show that cells behave predictably in zebrafish xenografts and therefore validate the model for use in cancer research and drug development. Cancer is an emergent property of rapidly dividing cells and their environment, and the environment controls much of the progression of the disease, as in the example of cancer-associated fibroblasts [127]. The zebrafish xenograft tests the compatibility between rapidly dividing cells and the available complement of molecular factors. If implanted tumour cells behave differently in the zebrafish xenograft, then it is likely due to the lack of recognizable niche characteristics or diffusible signals. The genetic differences between zebrafish and humans are defined, so these predictions are testable, as in the example of the MMP family of proteases. Conversely, when behaviour is conserved between xenograft models, then molecules and pathways that are absent or highly divergent are not directly involved. Better understanding these conserved and derived mechanisms in tumour biology is crucial to our knowledge of how cancer has evolved and how best to prevent and treat it.

## 5. Conclusions

The zebrafish xenograft is defended for its ability to replicate 2D and 3D culture results as well as, paradoxically, for its supposed superiority to them. The assumption that transplants will be more relevant than culture dish work has not been tested, though it is clear that there are significant differences introduced in gene expression [79]. Many reviews of the field exist that defend the use of zebrafish as a model for cancer research [4,5,6,7], and similar balanced criticisms exist for mouse models (for example, see [128]). We need continual assessments of the applications for all of the models we use in order to gauge the biological and clinical relevance of each. We need to maintain a balanced assessment of the strengths and weaknesses of our model systems, but we must also use a varied set of experiments.

The zebrafish xenograft has many advantages, but it is important that we remember the evolutionary context of this assay. The zebrafish assay is well placed to image the ultrastructure of implanted tumours and to begin to map molecular components, the activities of ECM-remodeling effectors, alterations of tissue architecture, vasculature and necrosis onto spatial patterns of cells, and ECM. Using the xenograft, we can ask questions about how cancer cells might begin to interact with the microenvironment at a metastatic location, though we learn less about the communication between a primary tumour and its microenvironment. There are zebrafish allograft models that can serve to image the interactions between the adaptive immune system and a growing tumour, but most xenograft work is designed to avoid the problem of the immune system. Nevertheless, we can examine the interactions between an implanted tumour and the non-adaptive immune system present in the zebrafish embryo.

The zebrafish xenograft, even more than the mouse xenograft, is performing an evolutionary experiment that could provide insights into the molecular components and networks of genes that are key for tumour growth, invasion, and ultimately metastasis. Many gene products, such as the MMPs, will not necessarily be represented in the microenvironment of the graft host. Using this absence, we can ask whether the elements that are required from stromal cells and other co-opted cell populations for these processes are available in the zebrafish host.

## Figures and Tables

**Table 1:** Matrix metalloproteinase (MMP) representation in zebrafish. The MMP family of proteases are unevenly represented in zebrafish, adapted from [105]. A single asterisk indicates one copy present in zebrafish, double asterisk indicates duplicates and dash indicates absence.

MMP	Zebrafish Representation	MMP	Zebrafish Representation
2	*	1	-
9	*	8	-
14	**	13	**
15	*	19	*
16	**	7	-
17	**	26	-
24	*	20	**
25	**	21	*
3	-	23	**
10	-	27	-
11	**	28	*
12	-		

## Supplementary Materials

The following are available online at www.mdpi.com/2073-4425/8/9/220/s1. Table S1: Keyword searches and literature survey counts by year for Figure 2.
